# Vitamin D Deficiency Is Associated with Increased Mortality and Seizure Risk After Nontraumatic Subarachnoid Hemorrhage: A Propensity Score-Matched Cohort Study

**DOI:** 10.3390/brainsci16050506

**Published:** 2026-05-08

**Authors:** Saketh Amasa, Parsa Radfar, Aiyana Adams, Asha Collier, Justin Buendia

**Affiliations:** 1 John Sealy School of Medicine, University of Texas Medical Branch, Galveston, TX 77550, USA; paradfar@utmb.edu; 2 Texas A&M Vasisht College of Medicine, Texas A&M University, College Station, TX 77843, USA; aiyanaa@tamu.edu; 3 Department of Public Health, University of Texas at Austin, Austin, TX 78712, USA; 4 School of Medicine, Boston University, Boston, MA 02118, USA

**Keywords:** vitamin D, subarachnoid hemorrhage, mortality

## Abstract

Background: Subarachnoid hemorrhage (SAH) is associated with high morbidity and mortality despite advances in neurocritical care. Vitamin D plays a role in immune modulation, endothelial function, and neuroprotection; however, its impact on outcomes following SAH remains poorly defined. We evaluated the association between low vitamin D status and clinical outcomes in patients with nontraumatic SAH. Methods: We conducted a retrospective propensity score-matched cohort study using the TriNetX Research Network database. Adult patients with nontraumatic SAH and at least one recorded serum 25-hydroxyvitamin D level obtained within 3 months on or before diagnosis were included. The low vitamin D cohort was defined as 0–20 ng/mL, and the comparator cohort as 20–40 ng/mL. Cohorts were matched 1:1 using propensity scores adjusted for demographic and clinical covariates, including chronic kidney disease, liver disease, osteoporosis, and intestinal malabsorption. The primary outcome was 30-day all-cause mortality. Secondary outcomes included seizures, hydrocephalus, cerebral edema, and external ventricular drain placement. Results: After matching, 2314 patients were included in each cohort. Thirty-day mortality occurred in 9.3% of patients in the low vitamin D cohort and 7.6% of patients in the comparator cohort (hazard ratio [HR] 1.229; 95% CI, 1.006–1.503; *p* = 0.043). Seizures were more frequent in the low vitamin D cohort (8.6% vs. 6.9%; odds ratio [OR] 1.274; 95% CI, 1.026–1.581; *p* = 0.028). Hydrocephalus was also more common among patients with low vitamin D (5.1% vs. 3.9%; OR 1.328; 95% CI, 1.003–1.758; *p* = 0.047). No significant differences were observed in cerebral edema or external ventricular drain placement. Conclusions: Low vitamin D status was associated with increased short-term mortality, seizure incidence, and hydrocephalus following nontraumatic SAH. These findings suggest that vitamin D status may represent a potential prognostic biomarker warranting prospective investigation.

## 1. Introduction

Subarachnoid hemorrhage (SAH) is a devastating neurological condition associated with substantial morbidity and mortality, accounting for approximately 5% of all strokes but contributing disproportionately to stroke-related deaths and long-term disability [1,2,3]. Despite advances in neurosurgical and critical care management, outcomes following SAH remain highly variable, with mortality rates approaching 30–40% and many survivors experiencing persistent neurological deficits [1,4,5,6].

Vitamin D plays a critical role in immune modulation, endothelial function, and neuroprotection [7,8,9]. Beyond its traditional role in bone metabolism, vitamin D has been implicated in the regulation of inflammatory cascades, oxidative stress, and vascular integrity, mechanisms highly relevant to secondary brain injury following SAH [7,8,10]. Prior studies have demonstrated associations between vitamin D deficiency and adverse outcomes in ischemic stroke, traumatic brain injury, and cardiovascular disease [11,12,13]. However, limited data exist regarding the impact of vitamin D status on clinical outcomes following SAH [14].

Given vitamin D’s potential neuroprotective properties and the high prevalence of deficiency in hospitalized populations, elucidating its relationship with SAH outcomes may identify a clinically relevant prognostic factor with potential therapeutic implications [9,15].

We hypothesized that vitamin D deficiency would be associated with increased mortality and higher rates of neurological complications in patients with nontraumatic SAH [1,14].

## 2. Methods

### 2.1. Study Design and Setting

We conducted a retrospective propensity score-matched cohort study to evaluate the association between vitamin D deficiency and clinical outcomes in patients with nontraumatic subarachnoid hemorrhage (SAH). This study utilized data from the TriNetX Research Network (Cambridge, MA, USA), a federated health research database that provides deidentified electronic health record (EHR) data from approximately 69 healthcare organizations across the United States, including academic tertiary care centers and large healthcare systems. The TriNetX platform aggregates real-world clinical data encompassing diagnoses, procedures, laboratory values, medications, and mortality outcomes.

All data within the TriNetX platform are deidentified and compliant with the Health Insurance Portability and Accountability Act (HIPAA); therefore, this study was exempt from institutional review board oversight. The data query was conducted on 28 April 2026. This study was reported in accordance with the Strengthening the Reporting of Observational Studies in Epidemiology (STROBE) guidelines.

### 2.2. Participants

#### 2.2.1. Study Population and Inclusion Criteria

Adult patients diagnosed with nontraumatic subarachnoid hemorrhage were identified using the International Classification of Diseases, Tenth Revision, Clinical Modification (ICD-10-CM). Patients were eligible for inclusion if they: (1) were aged ≥18 years at the time of SAH diagnosis; (2) received care between 1 January 2010, and 28 March 2026; (3) had at least one recorded serum calcidiol (25-hydroxyvitamin D) level obtained within 3 months on or before the index SAH diagnosis; and (4) had available follow-up data for outcome assessment. The index event was defined as the earliest recorded diagnosis of SAH during the study period.

#### 2.2.2. Exclusion Criteria

Patients were excluded if they: (1) had traumatic subarachnoid hemorrhage; (2) lacked a qualifying vitamin D measurement within the prespecified pre-index exposure window; (3) had outcome events documented prior to the analysis window when applicable; or (4) had missing data on key covariates required for propensity score matching.

#### 2.2.3. Cohort Definition

Two cohorts were created based on serum calcidiol levels measured within 3 months on or before SAH diagnosis. The low vitamin D cohort included patients with levels of 0–20.0 ng/mL, and the comparator cohort included patients with levels between 20.0 and 40.0 ng/mL, consistent with the predefined TriNetX query criteria. Variable Definitions and Data Collection.

All variables were extracted from structured EHR fields within the TriNetX platform. Demographic variables included age (continuous), sex, and self-reported race and ethnicity. Baseline comorbidities were identified using ICD-10-CM codes and included diabetes mellitus (E08–E13), essential hypertension (I10), disorders of lipoprotein metabolism (E78), chronic kidney disease (N18), liver disease (K70–K77), osteoporosis (M81), intestinal malabsorption (K90), nicotine dependence (F17), alcohol-related disorders (F10), and obesity as defined by body mass index (BMI). BMI was calculated from recorded height and weight measurements and categorized as underweight (<18.5 kg/m^2^), normal weight (18.5–24.9 kg/m^2^), overweight (25.0–29.9 kg/m^2^), obese class I (30.0–34.9 kg/m^2^), obese class II (35.0–39.9 kg/m^2^), and obese class III (≥40.0 kg/m^2^). Smoking status and alcohol use disorders were defined using diagnostic codes recorded prior to or at the time of SAH diagnosis.

### 2.3. Outcomes

#### 2.3.1. Primary Outcome

The primary outcome was all-cause mortality assessed over a 30-day follow-up period following SAH diagnosis. Mortality data within the TriNetX platform are derived from multiple sources, including EHR documentation and linked death registry data, providing reliable ascertainment of vital status.

#### 2.3.2. Secondary Outcomes

Secondary outcomes were assessed during the follow-up period and included neurological complications commonly associated with SAH, including hydrocephalus, cerebral edema, seizures, and external ventricular drain placement. All outcome definitions were prespecified and based on established clinical coding practices.

### 2.4. Propensity Score Matching

To reduce confounding and balance baseline characteristics between cohorts, we performed 1:1 propensity score matching using a greedy nearest-neighbor algorithm without replacement and a caliper width of 0.1 pooled standard deviations. Propensity scores were estimated using multivariable logistic regression with vitamin D deficiency as the dependent variable.

Prespecified covariates included age (continuous), sex, race, ethnicity, diabetes mellitus, essential hypertension, disorders of lipoprotein metabolism, BMI category, smoking status, alcohol use disorders, chronic kidney disease, liver disease, osteoporosis, and intestinal malabsorption.

The TriNetX platform employs randomized row ordering to minimize selection bias during the matching process. Covariate balance was assessed using standardized mean differences (SMDs), with values < 0.1 considered indicative of adequate balance. Distribution of propensity scores before and after matching was examined to further evaluate matching quality.

### 2.5. Statistical Analysis

All analyses were conducted on the propensity score-matched cohorts. Categorical variables are presented as frequencies and percentages, and continuous variables as means with standard deviations. Baseline characteristics were compared using chi-square tests for categorical variables and Student’s *t*-tests for continuous variables.

Survival analysis for the primary outcome was performed using Kaplan–Meier curves with log-rank testing. Cox proportional hazards regression was used to estimate hazard ratios (HRs) with 95% confidence intervals (CIs). To evaluate whether temporal changes in clinical practice or vitamin D testing influenced the primary findings, we performed an era-stratified sensitivity analysis by repeating the mortality analysis separately among patients diagnosed during 2010–2015 and 2016–2026. Secondary outcomes were analyzed using logistic regression to estimate odds ratios (ORs) with corresponding 95% CIs. Absolute risk differences were calculated where appropriate.

All statistical tests were two-sided, and a *p*-value < 0.05 was considered statistically significant. Secondary outcome analyses were considered exploratory, and no adjustments were made for multiple comparisons. Missing data were handled using complete case analysis, and the extent of missingness was documented for all variables.

All analyses were performed using the integrated statistical tools within the TriNetX Analytics platform, which has been previously validated for large-scale, real-world observational research.

## 3. Results

### 3.1. Study Population and Baseline Characteristics

The study included two propensity score-matched cohorts of patients (Table 1)with nontraumatic subarachnoid hemorrhage (SAH): one with low vitamin D levels (serum calcidiol ≤ 20 ng/mL) and one with comparator vitamin D levels (20–40 ng/mL). After 1:1 propensity score matching, each cohort comprised 2314 patients (Table 1). Baseline demographic and clinical characteristics were well balanced after matching, with all standardized mean differences < 0.10.

The mean (SD) age was 58.8 (18.8) years in the low vitamin D cohort and 58.3 (21.6) years in the comparator cohort (*p* = 0.483). Female patients comprised 50.6% and 51.2% of the cohorts, respectively. Racial and ethnic distributions were comparable, including White race (65.5% vs. 66.2%), Black or African American race (18.7% vs. 18.4%), Hispanic or Latino ethnicity (8.6% vs. 8.4%), and Asian race (4.9% vs. 4.7%).

Clinical comorbidities were also balanced after matching, including diabetes mellitus (30.1% vs. 29.9%), hypertension (64.6% vs. 63.8%), dyslipidemia (44.9% vs. 43.0%), chronic kidney disease (22.2% vs. 21.7%), liver disease (18.0% vs. 17.7%), osteoporosis (7.6% vs. 8.0%), and intestinal malabsorption (1.6% vs. 1.9%). Body mass index distributions were similarly comparable between cohorts. Because cohort inclusion required a recorded pre-index vitamin D measurement, the matched study population represents a selected subset of patients with SAH who underwent laboratory testing before diagnosis.

### 3.2. Primary Outcome: 30-Day Mortality

At 30 days following SAH diagnosis, mortality occurred in 210 of 2263 patients (9.3%) in the low vitamin D cohort and 174 of 2280 patients (7.6%) in the comparator cohort (Table 2). Kaplan–Meier survival analysis demonstrated lower 30-day survival probability in the low vitamin D cohort (90.53% vs. 92.19%; log-rank *p* = 0.043). Low vitamin D status was associated with a significantly increased hazard of 30-day mortality (hazard ratio [HR] 1.229; 95% CI, 1.006–1.503; *p* = 0.043).

In era-stratified sensitivity analyses (Table 3), the direction of association between low vitamin D status and higher 30-day mortality remained consistent across both study periods. Among patients diagnosed during 2010–2015, 30-day survival probability was 92.95% in the low vitamin D cohort versus 95.88% in the comparator cohort (log-rank *p* = 0.148). Among patients diagnosed during 2016–2026, corresponding survival probabilities were 90.26% versus 91.82% (log-rank *p* = 0.111). Although these subgroup comparisons did not reach statistical significance, likely due to reduced sample size after stratification, the findings were directionally consistent with the primary analysis.

### 3.3. Secondary Outcomes

#### 3.3.1. Neurologic Complications

Seizures occurred in 200 patients (8.6%) in the low vitamin D cohort compared with 160 patients (6.9%) in the comparator cohort (risk difference 1.7 percentage points; OR 1.274; 95% CI, 1.026–1.581; *p* = 0.028) (Table 2).

Hydrocephalus developed in 118 patients (5.1%) in the low vitamin D cohort and 90 patients (3.9%) in the comparator cohort (risk difference 1.2 percentage points; OR 1.328; 95% CI, 1.003–1.758; *p* = 0.047) (Table 2).

Cerebral edema occurred in 115 patients (5.0%) in the low vitamin D cohort and 105 patients (4.5%) in the comparator cohort, with no statistically significant difference between groups (OR 1.100; 95% CI, 0.839–1.443; *p* = 0.490) (Table 2).

#### 3.3.2. Surgical Interventions

External ventricular drain (EVD) placement, after excluding patients with prior EVD before the outcome window, occurred in 38 of 2044 patients (1.9%) in the low vitamin D cohort and 26 of 2088 patients (1.2%) in the comparator cohort. This difference did not reach statistical significance (OR 1.502; 95% CI, 0.909–2.483; *p* = 0.110) (Table 2). Given the likelihood of undercapture of procedural outcomes in structured EHR data, this finding should be interpreted cautiously.

## 4. Discussion

In this large, multicenter retrospective cohort study using propensity score-matched data from over 4400 patients with nontraumatic subarachnoid hemorrhage (SAH), we found that vitamin D deficiency (serum calcidiol ≤ 20 ng/mL measured within 3 months on or before diagnosis) was associated with significantly worse short-term outcomes. Specifically, vitamin D-deficient patients demonstrated a 23% higher hazard of 30-day mortality compared to patients with normal vitamin D levels. This association persisted after rigorous adjustment for demographic and clinical covariates. Additionally, vitamin D deficiency was associated with a significantly higher incidence of seizures. Even modest increases in mortality risk may be impactful in SAH populations, where baseline mortality is high and modifiable risk factors are limited.

The increased mortality observed in the vitamin D-deficient cohort may be partially explained by vitamin D’s role in neurovascular homeostasis and inflammatory modulation [7,8,16,17]. Vitamin D exerts pleiotropic effects on endothelial integrity, immune regulation, and neuronal survival [7,8,18]. Experimental data demonstrate that vitamin D downregulates pro-inflammatory cytokines such as interleukin-6 and tumor necrosis factor-α while promoting anti-inflammatory signaling pathways [7]. In the context of SAH, where the initial hemorrhage is followed by a robust inflammatory cascade, blood–brain barrier disruption, and delayed cerebral injury, inadequate vitamin D levels may impair the brain’s capacity to mitigate secondary damage [16,19,20,21,22]. Dysregulated inflammatory signaling could amplify microvascular dysfunction, worsen cerebral perfusion, and increase vulnerability to delayed neurologic deterioration [16,19,23].

Vitamin D also plays a critical role in calcium homeostasis and neuronal excitability [18,24]. The higher rate of seizures observed in vitamin D-deficient patients (OR 1.274) may reflect altered neuronal membrane stability and increased cortical excitability in the setting of deficiency [24,25,26]. Vitamin D receptors are widely expressed in cortical and hippocampal neurons, and deficiency has been associated with heightened seizure susceptibility in both experimental and clinical settings [25,26,27,28]. In the acute post-SAH period, where cortical irritation and blood breakdown products already predispose to epileptiform activity, vitamin D deficiency may further lower seizure thresholds [29,30,31].

Interestingly, while point estimates for hydrocephalus were elevated in the vitamin D-deficient cohort, this was modestly more frequent in the low vitamin D cohort and reached nominal statistical significance, although the effect size was small and should be interpreted cautiously. Similarly, rates of cerebral edema, and external ventricular drain placement were comparable between groups. These findings suggest that the mortality signal associated with vitamin D deficiency may not be mediated through gross structural complications alone, but rather through more diffuse mechanisms such as systemic inflammation, immune dysregulation, metabolic vulnerability, or impaired recovery capacity [7,8,15,18]. Vitamin D deficiency has also been associated with endothelial dysfunction and impaired vascular reactivity, both of which are central to delayed cerebral ischemia following SAH [8,16,23].

Beyond neurologic mechanisms, vitamin D deficiency may reflect a broader state of physiologic frailty [32]. Hypovitaminosis D is common among older adults, individuals with chronic illness, and hospitalized populations [15,33,34]. It has been linked to immune dysfunction, impaired wound healing, sarcopenia, and increased susceptibility to infection [17,35,36,37]. Thus, vitamin D deficiency may function as a biomarker of diminished systemic reserve [17,18,32]. In the high-stress physiologic environment following SAH, reduced immunologic and metabolic resilience may translate into higher mortality even in the absence of overt structural complications [17,20,32].

These findings may have potential clinical implications. First, vitamin D status is easily measurable, widely available, and modifiable [38]. Supplementation is inexpensive and generally safe when administered appropriately. However, whether correction of deficiency improves outcomes in SAH remains unknown and warrants prospective study. Randomized controlled trials evaluating targeted vitamin D supplementation in neurocritical care populations could help clarify whether deficiency is merely a prognostic marker or a modifiable therapeutic target.

Our results align with prior literature demonstrating associations between vitamin D deficiency and worse outcomes in ischemic stroke, traumatic brain injury, and cardiovascular disease [12,39,40]. However, data specific to SAH remain limited. Smaller observational studies have suggested correlations between low vitamin D levels and increased stroke severity or poorer neurological recovery [39]. Our study expands upon this body of work by leveraging a large, multicenter cohort and robust matching methodology, enhancing external validity and generalizability across diverse healthcare systems.

We performed an era-stratified sensitivity analysis to assess whether the observed association was influenced by changes in clinical practice or vitamin D testing over time [41]. In both study periods (2010–2015 and 2016–2026), patients with low vitamin D demonstrated numerically lower 30-day survival than comparator patients, although subgroup analyses did not reach statistical significance after stratification. The persistence of numerically lower survival across both temporal eras suggests that the primary association was not solely driven by secular changes in vitamin D testing practices or SAH management over time.

This study has several limitations. First, its retrospective observational design cannot establish causality and remains vulnerable to residual confounding despite propensity score matching. Second, inclusion required a recorded vitamin D measurement within 3 months on or before SAH diagnosis, which likely selected a non-random subgroup of patients with differing healthcare utilization, comorbidity burden, or survivorship characteristics. Third, we were unable to assess hemorrhage severity scales, radiographic volume, delayed cerebral ischemia, vitamin D supplementation, or longitudinal correction of deficiency. Fourth, the study period spanned multiple years during which SAH management and vitamin D testing practices may have evolved. Fifth, reliance on structured EHR coding may introduce outcome misclassification and under-capture of some procedural or complication outcomes. Seizure ascertainment may also have been influenced by variation in institutional EEG monitoring practices, ICU length of stay, and documentation intensity. Centers that use continuous EEG are more likely to detect both clinical and subclinical seizures than centers relying primarily on bedside observation. Accordingly, the observed association between low vitamin D status and seizures may partially reflect differential detection rather than a purely biological effect. Finally, unmeasured confounders such as frailty, nutritional status, and socioeconomic factors may remain.

## 5. Conclusions

Low vitamin D status was associated with increased short-term mortality and higher seizure incidence in patients with nontraumatic subarachnoid hemorrhage. These findings suggest that vitamin D status may represent a potential prognostic biomarker warranting prospective investigation. Prospective studies are needed to determine whether early identification and correction of vitamin D deficiency can improve neurological recovery and survival in this high-risk population.

## Figures and Tables

**Table 1:** Baseline demographic and clinical characteristics of patients with nontraumatic subarachnoid hemorrhage stratified by vitamin D status after propensity score matching.

Characteristic	Low Vitamin D (≤20 ng/mL) (*n* = 2314)	Normal Vitamin D (20–40 ng/mL) (*n* = 2314)	*p* Value	Standardized Mean Difference
**Demographics**				
Age at index, mean (SD), y	58.8 (18.8)	58.3 (21.6)	0.483	0.021
Female sex, No. (%)	1170 (50.6)	1184 (51.2)	0.681	0.012
White race, No. (%)	1515 (65.5)	1531 (66.2)	0.620	0.015
Black or African American, No. (%)	433 (18.7)	425 (18.4)	0.762	0.009
Hispanic or Latino, No. (%)	198 (8.6)	195 (8.4)	0.874	0.005
Asian, No. (%)	114 (4.9)	109 (4.7)	0.731	0.010
**Medical History**				
Diabetes mellitus, No. (%)	697 (30.1)	691 (29.9)	0.847	0.006
Essential hypertension, No. (%)	1495 (64.6)	1476 (63.8)	0.560	0.017
Long-term anticoagulant use, No. (%)	443 (19.1)	446 (19.3)	0.911	0.003
Disorders of lipoprotein metabolism, No. (%)	1040 (44.9)	996 (43.0)	0.193	0.038
Chronic kidney disease, No. (%)	514 (22.2)	502 (21.7)	0.670	0.013
Liver disease, No. (%)	417 (18.0)	410 (17.7)	0.788	0.008
Osteoporosis, No. (%)	177 (7.6)	184 (8.0)	0.701	0.011
Intestinal malabsorption, No. (%)	37 (1.6)	45 (1.9)	0.373	0.026
**Body Mass Index**				
BMI, mean (SD), kg/m^2^	27.5 (7.2)	27.0 (6.7)	0.067	0.061
Underweight (<18.5), No. (%)	277 (12.0)	273 (11.8)	0.856	0.005
Normal weight (18.5–24.9), No. (%)	955 (41.3)	941 (40.7)	0.676	0.012
Overweight (25.0–29.9), No. (%)	1042 (45.0)	1034 (44.7)	0.813	0.007
Obese class I (30.0–34.9), No. (%)	761 (32.9)	758 (32.8)	0.925	0.003
Obese class II (35.0–39.9), No. (%)	399 (17.2)	396 (17.1)	0.907	0.003
Obese class III (≥40.0), No. (%)	245 (10.6)	229 (9.9)	0.438	0.023

Data are presented as No. (%) unless otherwise indicated. All standardized mean differences were <0.10, confirming adequate balance after matching.

**Table 2:** Clinical outcomes following nontraumatic subarachnoid hemorrhage according to vitamin D status in propensity score-matched cohorts.

Outcome	Low Vitamin D	Normal Vitamin D	Odds Ratio (95% CI)	*p* Value
**Primary Outcome**				
30-day Mortality	210/2263 (9.3%)	174/2280 (7.6%)	1.229 * (1.006–1.503)	0.043
**Secondary Outcomes**				
Seizures	200/2314 (8.6%)	160/2314 (6.9%)	1.274 (1.026–1.581)	0.028
Hydrocephalus	118/2314 (5.1%)	90/2314 (3.9%)	1.328 (1.003–1.758)	0.047
Cerebral edema	115/2314 (5.0%)	105/2314 (4.5%)	1.100 (0.839–1.443)	0.490
External ventricular drain	38/2044 (1.9%)	26/2088 (1.2%)	1.502 (0.909–2.483)	0.110

Data are presented as No. (%) unless otherwise indicated. All outcomes were analyzed using logistic regression. * Indicates Hazard ratio for Kaplan–Meier survival analysis.

**Table 3:** Era-stratified sensitivity analysis of 30-day mortality following nontraumatic subarachnoid hemorrhage according to vitamin D status.

Study Era	Low Vitamin D Cohort	Comparator Cohort	30-Day Survival Probability	30-Day Survival Probability	Log-Rank *p* Value
	Patients/Deaths	Patients/Deaths	Low Vitamin D	Comparator	
2010–2015	272/19	272/11	92.948%	95.878%	0.148
2016–2026	1671/159	1688/135	90.261%	91.816%	0.111

Low vitamin D defined as serum calcidiol 0–20 ng/mL measured within 3 months on or before diagnosis. Comparator cohort defined as serum calcidiol 20–40 ng/mL measured within 3 months on or before diagnosis.

## Data Availability

The data that support the findings of this study are available from the TriNetX research network but are not publicly available due to data use agreements with participating healthcare organizations. Access to the data may be obtained through a formal request to TriNetX, subject to institutional approval and applicable data use agreements.
